# Board meeting frequency and firm performance: examining the nexus in Nigerian deposit money banks

**DOI:** 10.1016/j.heliyon.2018.e00850

**Published:** 2018-10-11

**Authors:** Damilola Felix Eluyela, Olamide Oluwabusola Akintimehin, Wisdom Okere, Emmanuel Ozordi, Godswill Osagie Osuma, Simon Osiregbemhe Ilogho, Olufemi Adebayo Oladipo

**Affiliations:** a Department of Accounting and Finance, Landmark University, Omu Aran, Nigeria; b Department of Business Studies, Landmark University, Omu Aran, Nigeria; c Department of Economics, Accounting and Finance, Bells University of Technology, Ota, Nigeria; d Department of Accounting, Covenant University, Ota, Nigeria; e Department of Banking and Finance, Covenant University, Ota, Nigeria

**Keywords:** Business, Economics

## Abstract

The main aim of this study is to examine the impact of board meeting frequency on firm performance of deposit money banks in Nigeria. Data used for the study were spawned from annual reports of the deposit money banks listed on Nigeria stock exchange (NSE) market. We employed a panel regression to test the significant association amid variables. Our main empirical result shows a positive association amid board meeting frequency and firm performance. Although, our findings also show that board size was positive and not significant and firm size was negative and significant. The study recommended that management of banks should consider increasing their frequency of board meetings to at least four (4) meetings per year. This will allow the sampled deposit money banks to comply with the good governance code in Nigeria which states that companies must meet at least once per quarter.

## 1 Introduction

Corporate governance has been an issue of global concern as a result of the economic crisis and various financial frauds which lead to the failure of many companies in 2008. Corporate governance is seen as the tactic a company is being directed and controlled. This led to the agency problem due to the difference that exists amid ownership and control of companies. Mohamed et al. (2016) noted that the board of directors is the most imperative mechanism of corporate governance. The corporate board of directors plays integral and vital roles in every organization. The regular board meeting is of a great importance to the overall effectiveness and efficiency of every board. Every director is expected to attend all board meetings as this forms part of the requirement for re-nomination as a board member. Board meeting assists directors to be well equipped with information and with all development within the company.

Board meeting is an organized set up arranged to assemble directors on the board to discuss and address relevant issues relating to their prior experiences, current predicament, and forward looking matters as its relate to the company survival (going concern). Every resolution passed during this exercise is legal and become operational in the company. Board meeting frequency can be ascertained by the number of meetings held during a year by top level managers. The exercise serves as a salient medium for effective harmonization of opinion towards achieving firms overall objectives (goals).

Kakanda et al. (2016a) asserted that every company's survival and growth is assessed based on their corporate performance. There are however conflicting views as regards the measurement of corporate performance of every firm. Marn and Romauld (2012) see corporate performance as the way by which the limited resources are utilized efficiently and effectively in the achievement of the overall goal of the company. Berger and Patti (2002) on the other hand see the firm performance in the view of improving shareholders wealth. Corporate performance can be measured when shareholder attain satisfaction at the end of a financial year as compared to the start of a financial year. Financial ratios like return on asset (ROA), return on equity (ROE), and return on capital employed (ROCE) are used in determining the performance of a company. In achieving a better corporate performance, it's assumed that agency cost is reduced by the board of directors. This agency cost arises as a result of a conflict of interest between management and organizational goals (Mohamed et al., 2016).

There has been a consistent argument in literature as regards the essence of board meetings and performance of the board. This result in two different schools of thought. Some believe that for board members to effectively fulfil their function of strategy setting and management monitoring, there is a need for a frequent meeting from time to time (Vafeas, 1999). On the other hand, some asserted that frequent meeting leads to waste in managerial time, increase financial burden in terms of travelling expenses and sitting allowance given to board members. They conclude by stating that high board meeting frequency does not improve performance but the quality of such meetings does (Ntim and Osei, 2011; Taghizadeh and Saremi, 2013; Oyerinde, 2014).

The attention of academic literature has been on various board attributes influence on firm performance (Saibaba and Ansari, 2011; Dalton and Dalton, 2011; Nanka-Bruce, 2011; Rebeiz, 2015; Uwuigbe et al., 2018). These board attributes examined by previous researchers were board size, board independence, board diversity, board composition etc. However, there has been a dearth of literature on board process (which involves frequency and number of board meetings) as a variable for board attributes. To this background, the main objective of this paper is to examine the impact of board meeting frequency on firm performance. The study examined the Nigeria banking sector as an evidence.

The remaining part of this paper is as follows; the second section will examine the various literature reviews on board characteristics and firm performance, empirical and theoretical findings of previous research work on the study and hypothesis development. Section three will contain the summary of the methodology used which includes the method of data collection and method of data analysis. Section four includes the data collected, findings and discussion of findings. Lastly, section five is the concluding part of the study where recommendations, policy implications and suggestion for further studies will be discussed.

## 2 Background

The board meeting is a medium set up for deliberations on key issues and matters amongst board members in order to make certain important decisions for the progress and growth of any organization. The diligence of board members is often measured on the board meeting attendance frequency by each of the board members (Ghosh, 2007; Johl et al., 2015; Ilaboya and Obaretin, 2015). There is not slated governance law that determines the minimum amount of meetings to be attended by a board member as it were to the best of knowledge. This, therefore, means the control over board members individual diligence is internal and subjective to the chairman of that meeting. However, concerning the frequency of board meetings in general, it is reported that the fewer the meetings the better performance of the firm as a whole. Johl et al. (2015), in their study, revealed the negative relationship between board diligence and firm performance and one of their recommendations was that the meetings should be more important and less frequent. This is believed is said to be supported by Lorsch and MacIver (1989) (as cited in the work of Ilaboya and Obaretin, 2015), of which their observation was that frequent meetings lead to the diversion of an organization's time, energy and resources into less productive activities. This observation is also supported by (Mace, 1986; Useem, 2006; Johl et al., 2015; Ilaboya and Obaretin, 2015).

Ghosh (2007) opined that a statistically significant relationship exists between board diligence and firm performance. Nevertheless, the importance of board meetings cannot be overstressed because it is a vivid tool of governance. Certain banks have been ineffective in function in terms of oversight functions, as they tend to ratify management instructions and direction, even though it is obvious that such actions are against the rule of corporate governance. Such occurrence was as a result of the failure of the board committees to hold meetings for the performance of their meetings. This brings to mind, the necessary need for not only general board meetings but also committee meetings. Another key question that can be asked is the level of relevance of each of the committee meetings to board performance in general. Do some committees need more meeting frequencies than the other? This question is relative to the order of importance of each committee.

Johl et al. (2015) categorized board diligence as part of the key corporate governance mechanism that helps in guiding and advising the management towards the pursuit of shareholder interest amidst other control functions. The aforementioned study also detailed the regulation placed on Malaysian companies by regulators. The Malaysian code encourages regular board meetings and regular disclosure of details of frequency as well as member attendance. This is said to increase board effectiveness and also bring the board members into one mind by serving as a medium for disseminating salient information to all board members as regards the progress of the company. This has been proven by the works of Francis et al. (2012a) that revealed that boards with a low frequency of meeting performed poorly compared to the board with high frequency. This was further justified by the work of Ntim and Osei (2011) they conclude that board who meet more generate a higher level of performance than those who do not. With the view to further validate and revisit the two schools of thought concerning the effect of board meeting above, we aim at adding to knowledge by specifically concentrating our research on an African system of economy, laying focus on Nigeria as a scope of the study. Kakanda et al. (2016b) posited that board of directors play several and germane roles in an organization. Just as corporate governance is a compound word comprising of ‘corporate/corporation’ and ‘governance’ in literal terms corporate governance is defined as how a corporation is governed and the people to govern this corporation is the board of directors. This is why the board of directors is seen as key players in determining a firm's performance especially through their decisions from the outcomes of their meetings to be implemented in the organization.

Taghizadeh and Saremi (2013) examined a hundred and fifty (150) Malaysian public listed firm directors and their firm's performance. The result of their study suggested that high frequency of board meetings, a high percentage of independent non-executive directors decreases the return on equity (ROE), while female directors in terms of gender diversity on the board increase the return on equity (ROE). Johl et al. (2015) also studied Board characteristics and firm performances were they made use of annual report of seven-hundred (700) public listed firms in Malaysia. The result of their findings stated that board independence does not affect firm performance, while board financial expertise and board size are positively associated with the firm's performance.

In Nigeria, Oyerinde (2014) studied corporate governance and bank performance where he examined the degree to which corporate governance contributed to the Nigerian financial crisis especially in the banking sector between the years 2000–2010. He made use of panel data set to analyze the pre and post consolidation reforms of banks, using return on equity and net interest income as indicators of bank performance being the dependent variables and using variables such as the number of board members and related insider loan as indicators to measure corporate governance. The result of his findings averred that board size has a significant positive relationship to bank performance while insider loan is negatively related to bank performance. He further purported that insider loan which had a negative relationship to bank performance was the most detrimental consequence of lack of adequate corporate governance in the Nigerian banking industry.

### 2.1 Theory

Based on the premise that a board meeting is a dimension under board process which is a variable board attribute, the study, therefore, adopts the agency theory as a theoretical foundation. Agency theory is one of the most frequently adopted theoretical frameworks by finance and economics researchers in understanding the linkage between board features and firm value. The existence of Agency theory is based on the relationship between principals (shareholders) and agents (board members) which arise as a result of separation in ownership and control of a business enterprise, such that these shareholders appoint the board members to ensure the creation of a disciplined atmosphere, setting of timely and achievable strategic plan, and the effective control of the management team thereby ensuring firm performance which will lead to maximization of shareholder's value. In ensuring these, it is vital for the board of directors to have more meetings thereby increasing their capacity to advise, control and ensure discipline in an organization, so as to enhance corporate firm performance (Ntim and Osei, 2011). Corporate decisions can as well be effectively monitored through more frequent board meetings.

### 2.2 Hypothesis

Based on the mixed result from prior studies and theoretical development, the following hypothesis stated in the null form will be tested in section four of this study;

H_0_: Board meeting frequency has no significant relationship with the firm performance of deposit money banks in Nigeria.

## 3 Materials and methods

### 3.1 Samples

The study explore the association amid board meeting frequency and financial performance of registered deposit money banks in Nigeria. In other to attain this objective, the peculiarity of study panel regression was applied. However, the suitability of this method is based on the nature of the data harvested revolving on the combination of both time series and cross-sectional observation. Furthermore, the strength of this method is the ability to give room for freedom and reduced collinearity amidst constructs. The populace of this study covers the fifteen (15) registered deposit money banks on the Nigerian Stock Exchange (CBN, 2017). This research engages secondary data. The cradles of data were derived from the yearly reports and separate accounts of selected deposit money banks (Eluyela et al., 2018). The time frame for this study is eight years period (2011–2016).

Consequently to effectively provide a comprehensive information on aggregates, minimum and maximum observations for each constructs, the descriptive statistic was engaged. Thereafter the Pearson correlation test employed to ascertain the constructs are free from multicollinearity and to clearly explore any associations amidst constructs. This is in line with the work of Gujarati (2003) as cited by Okere et al. (2018) on the criterion for the absence of multicollinearity. However this work collaborates with their claim, the next and final step was to carry out the regression properly, but before it was done the Hausman test was administered to ensure model suitability (Fixed or Random effect Model).

The Table 1 below shows the measurement of variables used in this study. The independent variable is board meeting frequency while the dependent variable is firm performance. We used Tobin Q as our main measure of corporate performance. Tobin Q is a widely used proxy for operating performance in studies of corporate governance (Yermack, 1996; Vafeas, 1999; Anderson and Reeb, 2003; Ntim and Osei, 2011). For example, Gompers et al. (2003) conclude that firms with more shareholders right are better governed since they have a higher Tobin Q. Yermack (1996) analyzed board performance using Tobin Q while Anderson and Reeb (2003) examined the governance of family firms using Tobin Q. In this study, Tobin Q was used as a measure of financial performance because it measures both company market values and book values. This is the edge of Tobin Q to all other accounting performance measures that are solely based on book values measurement (Vafeas, 1999). This includes the return on capital employed (ROCE), return on investment (ROI), return on equity (ROE) etc.

**Table 1:** Summary of variables.

Variable name	Variable acronym	Variable type	Measurement
TOBIN Q	TOBQ	Dependent	The book value of total assets plus the market value of equity minus book value of equity divided by book value of total assets
Board meeting frequency	FBM	Independent	Natural logarithm of a number of the board meeting held throughout the financial year
Board size	BSIZE	Control	Total number of directors on the board
Firm size	FSIZE	Control	Natural logarithm of total asset

In line with Vafeas (1999), we included some control variables like BSIZE and FSIZE. The control variables were added to ensure the robustness of our results and alternative accounting based corporate performance.

### 3.2 Model

The model used in the study is described below. This model was adapted from the work of Collins and Kofi (2011). The Eq. (1) is computed implicitly as follows:

(1) Financial performance = F (board meeting frequency, board size, firm size)

where:

Financial performance is proxied as TOBQ

TOBQ = Book value of total assets plus the market value of equity minus book value of equity divided by book value of total assets.

This resulted in Eq. (2) shown below:

(2) TOBQ = f (FBMst-1, CONTROLSt-1)

In order to carry out various estimation test, the model is explicitly expressed in Eq. (3) as:

(3) TOBQ_it_ = α0 + FBMs_t-1_ + BSIZE_t-1_ + FSIZE_t-1_+ ε_t-1_

where;

TOBQ_it_ = *Tobin Q*

FBMs_t-1_ = *Board meeting frequency with respect to lag*

BSIZE_t-1_ = *Board size with respect to lag*

FSIZE_t-1_ = *Firm size with respect to lag*

α0 = *Coefficient of parameter*

ε_t-1_ = *Error term with respect to lag*

i = *denotes firms specific*

t = *denotes the deterministic time trend*

μ_t-1_ = *denotes the estimated residual with respect to lag*

## 4 Results and discussion

Table 2 below shows the descriptive statistics of all variables used to conduct the panel regression analysis for the study. TOBQ is between a minimum of 1.00 and a maximum of 1.89 with a mean of 1.14. However, FBM ranges from a minimum of 0.3 and a maximum of 1.11. This indicates that there is a mean of 1.0 (appx.) board meeting per year for the sampled banks. The control variables indicate a wide variations suggesting that the study samples have been sufficiently chosen and represent the entire population.

**Table 2:** Descriptive statistics.

Measurement	TOBQ	FBM	BSIZE	FSIZE
Mean	1.140053	0.763000	14.16327	10.53821
Median	1.132350	0.778200	14.00000	10.26265
Maximum	1.897300	1.113900	25.00000	12.49370
Minimum	1.005200	0.301000	7.000000	8.715600
Std. Dev.	0.087705	0.141907	3.028027	1.387070
Observations	98	98	98	98

From the Table 3 below, it can be seen that there is a positive relationship between TOBQ and FBM. This implies that for every 1% change in TOBQ, there will be a 1% change in board meeting frequency of Nigeria banks. However, a negative relationship exists between TOBQ & BSIZE and FSIZE.

**Table 3:** Correlation matrix.

	TOBQ	FBM	BSIZE	FSIZE
TOBQ	1.000000			
FBM	0.008773	1.000000		
BSIZE	−0.042155	0.228549	1.000000	
FSIZE	−0.050529	−0.087551	0.048311	1.000000

As a benchmark for multicollinearity, it can be seen that there is no correlation between the variables up to 80% as recommended in Okere et al. (2018). This shows the absence of multicollinearity between the variables applied in this study.

### 4.1 Regression analysis

In this section, the study employed panel data regression analysis to investigate the relationship between the frequency of board meeting and the firm's financial performance proxied by TOBIN Q. Tables 4 and 5 discussed the result of the panel regression analysis.

The Hausman test carried out in Table 4 is to determine which model is appropriate for the panel regression. The Hausman test rule is as follows:

**Table 4:** Hausman test.

Correlated random effects – Hausman test			
Test cross-section random effects			
Test summary	Chi-Sq. statistic	Chi-Sq. d.f.	Prob.
Cross-section random	16.843326	3	0.0008

If the P-value is statistically significant, accept the alternative hypothesis (Fixed Effect Model).

If the p-value isn't statistically significant, accept the null hypothesis (Random Effect Model).

From the analysis in Table 4 above, it is seen that the P-value (0.0008) < 5% significance level, so the null hypothesis is rejected and the alternative accepted which is a fixed effect model.

The result in the Table 5 above represents the panel regression of the dependent (TOBQ) and independent variables (FBM, BSIZE, and FSIZE). The result for the goodness of fit test as presented in table shows a coefficient of determination of R^2^ = 0.80 (80%) and adjusted R^2^ is 0.765 (77%); this shows that 77% of the total variation in the dependent variable (TOBQ) is explained by the independent variables (FBM, BSIZE, and FSIZE).

**Table 5:** Panel regression.

Dependent variable: TOBQ				
Method: Panel EGLS (Cross-section weights)				
Date: 04/22/18 Time: 14:52				
Sample: 2010 2016				
Periods included: 7				
Cross-sections included: 14				
Total panel (balanced) observations: 98				
Variable	Coefficient	Std. Error	t-Statistic	Prob.
FBM	0.014650	0.015913	0.920617	0.3600
BSIZE	0.000183	0.000757	0.241975	0.8094
FSIZE	−0.051561	0.015820	−3.259214	0.0016
C	1.669641	0.169474	9.851917	0.0000
	Effects specification			
Cross-section fixed (dummy variables)				
	Weighted statistics			
R-squared	0.803861	Mean dependent var		3.741848
Adjusted R-squared	0.765118	S.D. dependent var		2.557890
S.E. of regression	0.059928	Sum squared resid		0.290899
F-statistic	20.74831	Durbin-Watson stat		1.576425
Prob (F-statistic)	0.000000			

The p-value of the F statistics is 0.000000 which is significant at 5% explaining that the null hypothesis should be rejected. Consequently, the F-test results as depicted in the table indicates clearly that the fairness and non-biases of the model. It shows simultaneously that the independent variables altogether are significantly associated with the dependent variable. The high and statistically significant value of the F-statistic confirms the overall significance of the model and the predictive power of the independent variable. The Durbin Watson is 1.576425 which falls within the acceptable region and shows the presence of low auto-serial correlation which is common in time series data. This confirms the statistical reliability of the model.

Focusing on the relationship between board meeting frequency and financial performance, it can be seen from the table above that there exists a positive but non-significant relationship with a correlation coefficient value of 0.014650 and p-value of 0.3600. This implies that a unit increase in Board meeting frequency will lead to a 1.5% increase in the performance of deposit money banks in Nigeria. Due to the findings, the null hypothesis is accepted meaning that there is no significant relationship between board meeting frequency and financial performance of deposit money banks in Nigeria. This study findings resonate with the work of (Ntim and Osei, 2011; Francis et al., 2012a; Arosa et al., 2013; Taghizadeh and Saremi, 2013; Oyerinde, 2014) stating that board meetings only does not leads to better financial performance of the company but the quality of the meetings and implementation of timely decision taken during such meetings.

## 5 Conclusion

The study examines the relationship between board meeting frequency and performance of deposit money banks in Nigeria. This study further argues that since the corporate board of directors is the key players in determining the firm's performance through their decisions from the outcomes of their meetings, a regular board meeting is of great importance to the firms' overall effectiveness and efficiency. This claim is further supported by the findings of Francis et al. (2012b) and Ntim and Osei (2011). The findings provide empirical support for the agency theory, which suggests that when the board meets more frequently, this will increase their ability to effectively monitor, advise, scrutinize and create an atmosphere of discipline. This will improve their financial performance thereby achieving shareholders objective of maximizing their wealth. Also, the frequency of board meeting can be used as a measure of determining the activeness of a board. Board meeting also boards members to get the continuous report and take timely strategic decisions about the organization.

The study contributes to the existing knowledge on the impact of board attributes on the financial performance of listed firms. Literature attention has been on various board attributes influence on firm performance (Saibaba and Ansari, 2011; Rebeiz, 2015; Uwuigbe et al., 2018). These board attributes examined by previous researchers were board size, board independence, board diversity, board composition etc. This study contributed to the body of knowledge by examining the board meeting as a measure of board attributes. Our finding has some policy implication for banks and their board's members. Management of banks should consider increasing their frequency of board meetings to at least four (4) meetings per year. This will allow the sampled banks to comply with the minimum standard of good governance code of 2016 in Nigeria which states that companies must meet at least once per quarter (Financial Reporting Council of Nigeria code, 2016:17). This will allow members of the board to be properly informed about the activities of the organization in order to give contribute quota in strategic decision making. Also, the quality of these meetings should be examined over time to ensure that there is no room for an idle time.

The research work has some limitations. First, the data used for this study were generated from listed sampled deposit money banks in Nigeria. This will limit the generalization of our findings. Future researchers can examine the relationship between board meeting frequency and firm performance for companies listed in other sectors of the Nigeria Stock Exchange market.

## Declarations

### Author contribution statement

Damilola F. Eluyela, Emmanuel Ozordi: Conceived and designed the analysis; Contributed analysis tools or data; Wrote the paper.

Olamide O. Akintimehin, Wisdom Okere: Analyzed and interpreted the data; Contributed analysis tools or data; Wrote the paper.

Godswill O. Osuma, Simon O. Ilogho, Olufemi A. Oladipo: Contributed analysis tools or data; Wrote the paper.

### Funding statement

This work was supported by Landmark University.

### Competing interest statement

The authors declare no conflict of interest.

### Additional information

No additional information is available for this paper.
